# Design, synthesis, and spasmolytic activity of thiophene-based derivatives via Suzuki cross-coupling reaction of 5-bromothiophene-2-carboxylic acid: their structural and computational studies

**DOI:** 10.3906/kim-1911-51

**Published:** 2020-10-26

**Authors:** Nasir RASOOL, Hafiz Mansoor IKRAM, Ammara RASHID, Nazia AFZAL, Muhammad Ali HASHMI, Muhammad Naeem KHAN, Ayesha KHAN, Imran IMRAN, Hafiz Muhammad Abdur RAHMAN, Syed Adnan Ali SHAH

**Affiliations:** 1 Department of Chemistry, Government College University Faisalabad, Faisalabad Pakistan; 2 Department of Chemistry, University of Sahiwal, Sahiwal Pakistan; 3 Department of Chemistry, University of Education, Attock Pakistan; 4 School of Chemical and Physical Sciences, Victoria University of Wellington, Wellington New Zealand; 5 Department of Pharmacology, Faculty of Pharmacy, Bahauddin Zakariya University, Multan Pakistan; 6 Faculty of Pharmacy, Universiti Teknologi MARA Cawangan Selangor, Bandar Puncak Alam, Selangor Malaysia; 7 Atta-ur-Rahman Institute for Natural Products Discovery (AuRIns), Universiti Teknologi MARA Cawangan Selangor, Bandar PuncakAlam, Selangor Malaysia

**Keywords:** Suzuki cross-coupling reaction, 5-bromothiophene-2-carboxylic acid, density functional theory, spasmolytic activity

## Abstract

In the current research work, a facile synthesis of a series of novel thiophene-based derivatives of 5-bromothiophene-2-carboxylic acid ( **1** ) have been synthesized. All analogs ( **5a** - **5e** , **10a** - **10f** ) were obtained from the coupling reaction of 5-bromothiophene-2-carboxylic acid ( **1** ) and different arylboronic acids with moderate-to-good yields under controlled and optimal conditions. The structures of the newly synthesized compounds were characterized through spectral analysis and their spasmolytic activity, and most of the compounds exhibited potentially good spasmolytic effect. Among the synthesized analogs, compound phenethyl 5-(3,4-dichlorophenyl)thiophene-2-carboxylate ( **10d** ) particular showed an excellent spasmolytic effect with an EC _50_ value of 1.26. All of the compounds were also studied for their structural and electronic properties by density functional theory (DFT) calculations. Through detailed insight into frontier molecular orbitals of the compounds and their different reactivity descriptors, it was found that the compounds **10c** and **5c** are the most reactive, while **10a** is the most stable in the series. Furthermore, compounds **10c** and **5c** showed a very good NLO response with the highest *β* values.

## 1. Introduction

In the last several decades, the palladium-catalyzed Suzuki cross-coupling reaction has made a remarkable contribution to synthetic chemistry due to the formation of carbon-carbon bonds [1]. This is a versatile synthetic transformation leading to the synthesis of biaryl derivatives. Generally, this reaction is preferred over others due to several reasons, such as commercial availability of the starting material, mild reaction condition, and a large number of functional group tolerance. This reaction can be carried out in aqueous media, and produce nontoxic by-products which can be eliminated without difficulty [2]. The thiophene-based moieties have been used as starting precursors for the formation of several important products because of their availability and stability towards direct cross-coupling reaction [3–5]. The synthesis of thiophene-based compounds has aroused tremendous interest recently since they can be synthesized easily and exhibit biological activities. In the literature survey, the thiophene derivatives were found to exhibit several biological activities, including analgesic [6], antitumor [4], diabetes mellitus [7], antimicrobial [8], antiallergic [9], anticonvulsant [10], anti-HIV/AIDS inhibitor [11], antitubercular [12], BACE1 inhibitors [13], and antidepressant [14]. Also, they have shown numerous applications in energy storage appliances [15], optoelectronics, conductivity-based sensors, biodiagnostics, azo dyes [16], and non-linear optics [17,18]. Recently, we have reported the preparation and biological applications of several thiophene-based derivatives [3,4,18,19]. In particular, Ikram et al. [3,4] described the synthesis and biological significance of several thiophene-based derivatives with moderate-to-good yield. The biological significance of thiophene-based compounds encouraged us to synthesize some new thiophene derivatives and study their biological activities.

Hence, in the search of new biologically active agents, we report herein a facile synthesis of thiophene-based derivatives with moderate-to-good yields. The present work aimed to prepare several analogs of pentyl 5-bromothiophene-2-carboxylate ( **5a** - **5e** ) and phenethyl 5-bromothiophene-2-carboxylate ( **10a** - **10f** ), using 5-bromothiophene-2-carboxylic acid( **1** ) and different arylboronic acids through the Suzuki cross-coupling reactions. All these compounds were screened for their possible spasmolytic activity for biological characterization. The spasmolytic (antispasmodic) effect of drugs is generally used to suppress the excessive smooth muscle spasms, which are responsible for cramping and discomfort in the abdominal area and caused by multiple conditions. Such spasms affect the gastrointestinal, biliary, or genitourinary tract. The antispasmodic drugs are used to reduce the smooth muscle contractility, and for the treatment of irritable bowel syndrome (IBS) and biliary colic [20]. We also studied the structural and electronic properties of the synthesized derivatives using density functional theory (DFT). The structures of the synthesized compounds were characterized through EI-MS, ^1^ H NMR, and ^13^ C NMR spectral data.

## 2. Results and discussion

### 2.1. Chemistry

**Scheme 1:**
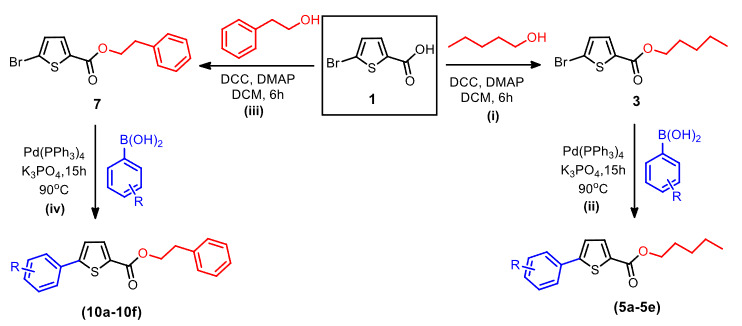
(i). Synthesis of **3: 1** (4 g, 19.0 mmol), DCM (200 mL), amyl alcohol (3 equiv.), DCC (1.1 equiv.), DMAP (0.05 equiv.), 30 °C. (ii). Synthesis of derivatives **5a** - **5e** : (iia). Dry toluene (5 mL), 3 (0.17 g, 0.61 mmol), arylboronic acid (0.61 mmol), Pd(PPh _3_ ) _4_ (5 mol%), potassium phosphate (1.28 mmol), 90 °C, 16 h reflux. ( ***iib*** ). 1,4-dioxane/H _2_ O (5 mL), 3 (0.17 g, 0.61 mmol), arylboronic acid (0.61 mmol), Pd(PPh _3_ ) _4_ (5 mol%), potassium phosphate (1.28 mmol), 90 °C, 16 h reflux. (iii). Synthesis of **7: 1** (6 g, 29 mmol), DCM (200 mL), 2-Phethanol (3 equiv.), DMAP (0.05 equiv.), DCC (1.1 equiv.), 30 °C. (iv). Synthesis of derivatives **10a** - **10f** : (iva). Dry toluene (5 mL), 3 (0.26 g, 0.64 mmol), arylboronic acid (0.64 mmol), Pd(PPh _3_ ) _4_ (5 mol%), potassium phosphate (1.28 mmol), 90 °C, 16 h reflux. (ivb). 1,4-dioxane/H _2_ O (5 mL), 3 (0.26 g, 0.64 mmol), arylboronic acid (0.64 mmol), Pd(PPh _3_ ) _4_ (5 mol%), potassium phosphate (1.28 mmol), 90 °C, 16 h reflux.

In the present research work, we started with the commercially available thiophene-based compound 5-bromothiophene-2-carboxylic acid ( **1** ), which was first esterified according to the previously reported method [21]. In this method, compound **1** was prepared to react with amyl alcohol to give an ester pentyl 5-bromothiophene-2-carboxylate (3) in 75% yield. In the above reaction, N,N’-dicyclohexylcarbodiimide (DCC) has taken part as a coupling reagent while 4-dimethylaminopyridine (DMAP) was used as a catalyst. The reaction was carried out at 30 oC, and dichloromethane (DCM) was taken as a solvent. The reaction is depicted in scheme 1 as (i). Ali et al. described one-pot synthesis of a wide range of 4-arylthiophene-2-carbaldehyde derivatives with good yields from 4-bromothiophene-2-carbaldehyde by using the Suzuki cross-coupling reaction [19].

**Figure 1:**
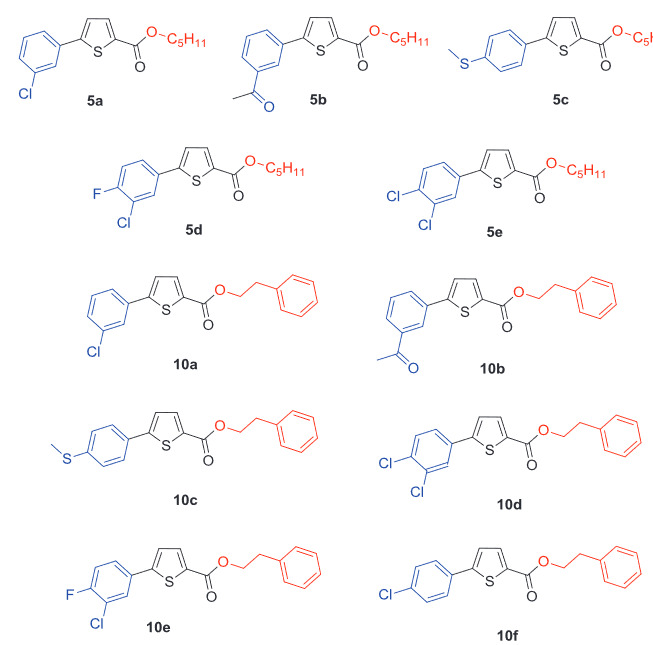
Synthesis of the thiophene-based derivatives ( **5a** - **5e** , **10a** - **10f** ).

To investigate the palladium-catalyzed Suzuki cross-coupling reaction of **1** , we designed a facile approach under optimized conditions. We focused on the synthesis, biological applications and structure, and electronic evaluation of the new thiophene derivatives. For this purpose, the esterified product **3** was treated with several arylboronic acids, leading to the formation of pentyl 5-arylthiophene-2-carboxylate derivatives ( **5a** - **5e** ). These derivatives were synthesized through the Suzuki cross-coupling reaction in the presence of tetrakis (triphenylphosphine) palladium (0) as a catalyst at 90 °C. As a result, the combination of pentyl 5-bromothiophene-2-carboxylate with several arylboronic acids produced pentyl 5-arylthiophene-2-carboxylate derivatives successfully ( **5a** - **5e** , Figure 1). The synthesis of **5a** - **5e** is described in Scheme 1 as (ii). To the best of our knowledge, the synthesis of pentyl 5-arylthiophene-2-carboxylate from 1 through Suzuki cross-coupling reaction has not been reported elsewhere. To study the effect of the solvent on the yield of the newly synthesized products, we carried out these coupling reactions in dry toluene as well as 1,4-dioxane along with water.

The results of this study are shown in Table 1. The results revealed that the newly synthesized thiophene-derivatives, **5a** , **5b** , **5c** , **5d** and **5e** , were obtained in 71.5%, 75%, 80.2%, 65%, and 70.2% yields, respectively, when 1,4-dioxane and water were used as a solvent in 4:1 ratio. The possible cause of the greater yield might be the higher solubility of the arylboronic acid in aqueous 1,4-dioxane. The current study also showed that the moderate yields were obtained in dry toluene because the products **5a** , **5b** , **5c** , **5d** , and **5e** were obtained in 50.2%, 33%, 76.5%, 51.5%, and 52.7% yields, respectively.

We also synthesized phenethyl 5-bromothiophene-2-carboxylate 7) in 71% yield by the reaction of 1 and 2-Phethanol in the presence of DCC and DMAP by the use of Steglich esterification reaction. Later on, the esterified product 7 was reacted with several arylboronic acids, leading to the formation of the corresponding phenethyl 5-arylthiophene-2-carboxylate derivatives ( **10a** - **10f** , Figure 1). The Suzuki cross-coupling reactions of 7 with several arylboronic acids produced thiophene-based analogs successfully (Table 2, Figure 1). The synthesis of the newly thiophene-based derivatives ( **10a** - **10f** ) is described in Scheme 1 as (iv). These reactions were also carried out in dry toluene as well as in aqueous 1,4-dioxane. The products **10a** , **10b** , **10c** , **10d** , **10e** , and **10f** were obtained in 68%, 66%, 69%, 56%, 55%, and 63%, respectively, in dry toluene. On the other hand, the products **10a** , **10b** , **10c** , **10d** , **10e** , and **10f** were obtained in 75%, 68%, 72%, 65%, 67%, and 64%, respectively, when a combination of organic solvent 1,4-dioxane and water was used as a solvent.

The current research revealed that the nature of the solvent has affected the yields of the final products. Consequently, we found that the combination of 1,4-dioxane and water produced better yields compared to dry toluene as previously reported [22].

**Table 1:** Synthesis of the pentyl 5-arylthiophene-2-carboxylates ( **5a** - **5e** ).

Entry	Arylboronic acid	Product	% yield in dry toluene	% yield in1,4-dioxane/water
1	3-chlorophenylboronic acid	**5a**	50.2	71.5
2	3-acetylphenylboronic acid	**5b**	33.0	75.0
3	4-(methylthio)phenylboronic acid	**5c**	76.5	80.2
4	3-chloro-4-flourophenylboronic acid	**5d**	51.5	65.0
5	3,4-dichlorophenylboronic acid	**5e**	52.7	70.2

**Table 2:** Synthesis of the phenethyl 5-arylthiophene-2-carboxylates ( **10a** - **10f** ).

Entry	Arylboronic acid	Product	% yield in dry toluene	% yield in1,4-dioxane/water
1	3-chlorophenylboronic acid	**10a**	68	75
2	4-acetylphenylboronic acid	**10b**	66	68
3	4-(methylthio)phenylboronic acid	**10c**	69	72
4	3,4-dichlorophenylboronic acid	**10d**	56	65
5	3-chloro-4-flourophenylboronic acid	**10e**	55	67
6	4-chlorophenylboronic acid	**10f**	63	64

### 2.2. Spasmolytic activity

To study the spasmolytic effect of the drugs, an isolated tissue of the intestine of a rabbit is used [23]. Drugs having antispasmodic effects usually show a relaxant effect if applied to spontaneously contracting the isolated pieces of the intestine or spasmogen treated intestine. In this study, the test compounds were applied to the isolated piece of the duodenum which was pretreated with a spasmogen (High-K ^+^ ). The effect was measured as the percentage of the induced contractions just before the administration of the test substance. Almost all the newly synthesized thiophene derivatives showed the spasmolytic effect. Compounds **5a** , **5b** , **5c** , and **5d** caused the complete relaxation of High-K ^+^ induced contractions with EC _50_ values of 4.21 µM with 95% CI (2.74–6.35), 7.09 µM with 95% CI (5.03–10.08), 1.39 µM with 95% CI (0.94– 2.02), and 11.8 µM with 95% CI (8.68–16.43). Compound **5e** caused partial relaxation of the induced contractions. Furthermore, compound 3, which was Suzuki cross-coupling reactant, did not show any response. This might have been due to the absence of an aromatic phenyl ring with a thiophene group.

The evaluation of the derivatives of the phenethyl 5-bromothiophene-2-carboxylate also showed spasmolytic activity. The results showed that when these compounds were tested on K ^+^ (K-80 mM) induced contractions in isolated rat duodenum, the test compounds named **10b** , **10c** , **10d** , and **10e** showed complete relaxation with respective EC _50_ values of 2.13 µM with 95% CI (1.32–3.46), 3.14 µM with 95% CI (1.68–6.06), 1.26 µM with 95% CI (0.64–2.67), and 2.89 µM with 95% CI (2.02–4.06). Compounds **10a** and **10f** did not show any response. The results are represented in Table 3 according to their potency from top to bottom. The compounds with high potency have a lower EC _50_ value while the compounds with low potency have higher EC _50_ values.

The high concentration of potassium High-K ^+^ (K-80mM) in the extracellular fluid may result in the opening of voltage-operated calcium channels and leads to the contraction of smooth muscles. The compounds which cause the relaxation of K-80mM induced contractions in smooth muscles are considered as calcium channel antagonists [24–26]. The results of the present study have shown that the spasmolytic activity of thiophene derivatives may be due to the blockage of calcium channels. It has been reported previously that the thiophene derivatives possessed calcium channel blocking activity [27,28]. The replacement of the hydroxyl group with the phenyl group may enhance the spasmolytic activity of thiophene derivatives. This blockage of calcium channels is receptor linked or direct, and requires highly sophisticated and extensive research.

**Table 3:** Spasmolytic effect of SB series on High-K ^+^ induced contractions in isolated rat duodenum, expressed as % age of control response before the administration of any test compound.

Treatment	Dose [µM]								EC _50_
	Control	0.1	0.3	1.00	3.00	5.00	10.00	30.00	
High-K ^+^ **10d**	100	92.0 ± 1.72	67.7 ± 8.39	51.4 ± 6.42	0	0	0	0	1.26
High-K ^+^ **5c**	100	90.4 ± 1.70	74.4 ± 1.84	60.3 ± 3.38	29.7 ± 4.93	0	0	0	1.39
High-K ^+^ **10b**	100	95.8 ± 2.19	80.8 ± 4.04	60.3 ± 4.21	35.2 ± 2.98	0	0	0	2.13
High-K ^+^ **10e**	100	97.7 ± 0.93	81.4 ± 1.80	64.6 ± 2.33	49.2 ± 3.49	33.2 ± 1.67	20.5± 2.41	0	2.89
High-K ^+^ **10c**	100	92.0 ± 1.77	78.4 ± 2.98	65.6 ± 3.35	43.5 ± 4.18	0	0	0	3.14
High-K ^+^ **5a**	100	92.4 ± 1.65	80.0 ± 2.52	73.4 ± 2.50	57.3 ± 3.29	33.4 ± 4.77	0	0	4.21
High-K ^+^ **5b**	100	91.6 ± 2.12	81.1 ± 2.17	74.9 ± 4.19	62.8 ± 3.17	46.9 ± 1.94	22.2 ± 1.87	0	7.09
High-K ^+^ **5d**	100	99.7 ± 0.27	93.7 ± 2.31	83.3 ± 1.89	77.5 ± 1.59	55.7 ± 4.28	33.9 ± 4.95	0	11.8
High-K ^+^ **5e**	100	100	100	97.9 ± 2.03	92.2 ± 4.51	87.9 ± 4.40	81.4 ± 4.23	77.0 ± 4.15	-
Data represented as mean ± SEM of 3–5 individual experiments.									

### 2.3. Computational studies

Density functional theory (DFT) calculations have been performed for all the molecules, using Gaussian 09 (Revision D.01) [29] software tool. For all the calculations, Adamo’s hybrid [30] version of Perdew, Burke, and Ernzerh of functional (PBE0) [31,32] has been used. Grimme’s empirical dispersion correction (D3) with Becke-Johnston damping (D3BJ) [33–35] has been applied in all the calculations. The basis set used in all the calculations was Ahlrich’s triple z basis set def2-TZVP[36], which was aided by the polarizable continuum model (PCM) with the integral equation formalism variant (IEFPCM) [37–43] for solvation modelling. Cramer and Truhlar’s [44] SMD parameter has been used as implemented in Gaussian 09 [29] to model the effects of 1,4-dioxane as a solvent. The structures after optimization have been confirmed to be minima on the potential energy surface by calculating their force constants showing no imaginary modes. The force constants have been calculated at the same level of theory, i.e. PBE0-D3BJ/def2-TZVP/SMD _1,4-dioxane_ . Nonlinear optical (NLO) properties calculations have been performed at the same level of theory as described above. For visualization and 3D images, GaussView 5.0.9 and CYLview [45] tools have been used.

**Figure 2:**
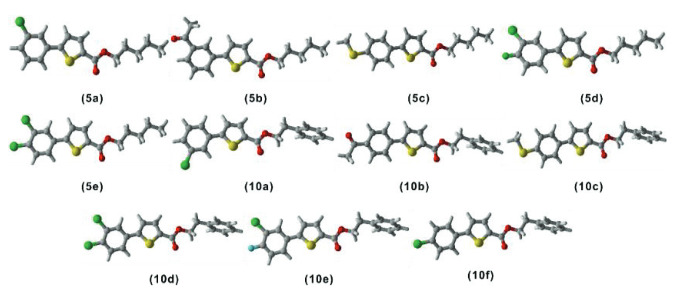
Optimized structures of all the compounds ( **5a** - **5e** , **10a** - **10f** ) at PBE0-D3BJ/def2-TZVP/SMD _1,4-dioxane_ level of theory. In 3D models, white represents hydrogens, green is for chlorine atoms, light blue color represents fluorine atoms, yellow color represents sulphur, grey color represents carbon, red color is for oxygen, and blue color shows nitrogen atoms.

All the thiophene derivatives ( **5a** - **5e** and **10a** - **10f** , Figure 2) have been modeled in GaussView followed by a geometry optimization at the PBE0-D3BJ/def2-TZVP/SMD _1,4-dioxane_ level of theory, using Gaussian 09 software.

### 2.4. FMO analysis

**Figure 3:**
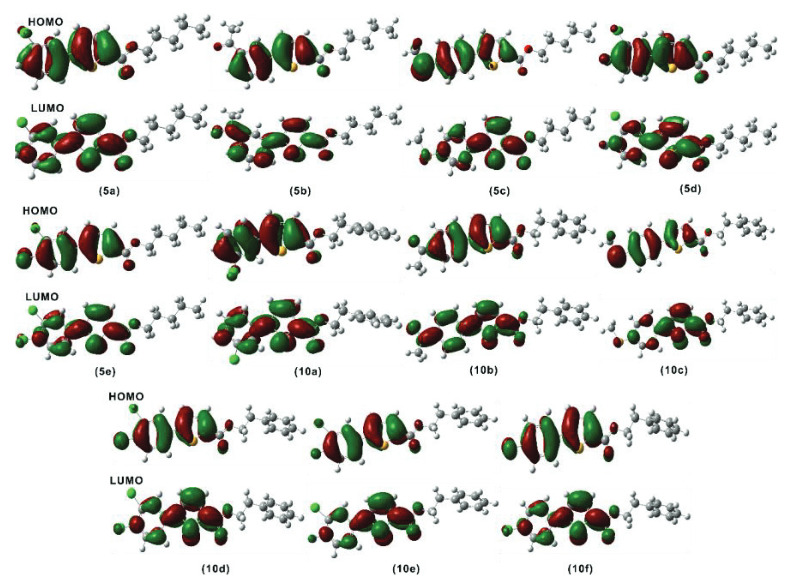
A presentation of all the frontier orbitals of all the molecules ( **5a** - **5e** , **10a** - **10f** ) calculated at PBE0-D3BJ/def2-TZVP/SMD _1,4-dioxane_ level of theory.

Frontier molecular orbitals (FMO) calculations were not as easy as they have become with the development in DFT and modern computers. Today, one can get a deeper understanding of the electronic properties and reactivity of molecules with the help of FMO calculations [46]. The main transitions of electrons that occur in molecules are usually between FMOs, and the gap between these two (HOMO-LUMO gap) provides useful information regarding kinetics and reactivity of a compound [47]. The wider this gap is, the lesser the reactivity is and vice versa. FMO information has been extracted from optimized geometries, and a plot of FMOs of all the molecules under study is shown in Figure 3.

Table 4 shows the HOMO-LUMO gap values (DE) of the title compounds. The DE values lie in a narrow range for all these molecules, i.e. 4.12–4.70 eV. This narrow range means that the reactivity difference for these compounds ( **5a** - **5e** , **10a** - **10f** ) is not much. According to these values, compound **10a** is the least reactive (DE = 4.70 eV) while **10c** and **5c** are the most reactive ones.

The isodensity dispersion in the FMOs of all the molecules ( **5a** - **5e** , **10a** - **10f** ) has shown a very similar trend for all of the compounds. The isodensity is mainly located on the thiophene ring and the aromatic ring attached to it for both HOMO and LUMO with no isodensity on the aliphatic chain. This uniformity in isodensity makes the compounds stable and less reactive.

### 2.5. Nonlinear optical properties

Nonlinear optical (NLO) properties exist due to the interaction of light with a nonlinear molecule. These nonlinear molecules have got considerable attention in the recent past due to their applications in optics, sensors, imaging, and medicine [48–50]. DFT calculated hyperpolarizability ( *β* ) values of organic compounds represents their ability to have electron push and pull in them. The higher the *β* values are, the greater the NLO response of the molecule is due to greater electron push and pull.

**Table 4:** Energies of HOMO, LUMO, and the gap between HOMO-LUMO. All the HOMO and LUMO energies are given in eV. Hyperpolarizability (β) values are given in Hartrees.

Compound	E _*HOMO*_	E _*LUMO*_	HOMO-LUMO gap	Hyperpolarizability
**5a**	–6.59	–1.90	4.69	1171.84
**5b**	–6.60	–1.97	4.63	845.36
**5c**	–5.85	–1.72	4.13	7106.78
**5d**	-6.56	-1.89	4.68	1974.20
**5e**	–6.57	–2.00	4.57	2083.48
**10a**	–6.61	–1.91	4.70	1122.71
**10b**	–6.63	–2.24	4.38	2005.11
**10c**	–5.87	–1.75	4.12	7672.72
**10d**	–6.59	–2.02	4.58	2328.68
**10e**	–6.58	–1.91	4.67	2209.09
**10f**	–6.47	–1.90	4.57	2630.26

NLO properties of the compounds under study have been computed using DFT. The compounds under study have phenyl rings that behave as electron acceptors, and thiophene and other substituents act as electron acceptors, thus causing an electron flow through the system. It is observed that strong electron donors on the phenyl rings increase the *β* values due to an increased flow of electrons through the system and vice versa. Due to these facts, SCH _3_ groups on the *para* position of the phenyl ring joined to thiophene backbone act as strong activators which put compounds **10c** and **5c** on top of the others for their NLO responses whose values are very high (β values of 7672 and 7106 au, respectively; Table 4). These two compounds can be good candidates for further research, having strong NLO response to find applications as optical materials.

### 2.6. Conceptual DFT reactivity descriptors

There are some important descriptors — including ionization potential ( *I* ), electron affinity ( *A* ), chemical hardness ( *ƞ* ), and electronic chemical potential ( *μ* )—that can be used to study the chemical reactivity of a compound. DFT calculations also provide this information to compute these reactivity descriptors. These reactivity descriptors have been calculated based on Koopman’s theorem, and the details of these calculations have been described previously [51]. The values of all these are given in Table 5.

As shown in the FMO analysis earlier, the chemical hardness is also supporting the results. Compounds **10c** and **5c** have the lowest values of ƞ, which makes the flow of electrons easier in these species, thus making them the most reactive in the series. Similarly, electrochemical potential (μ) represents the tendency of a specie to distribute itself equally in the solution in a container, so asmaller value of μ will lead to more reactivity, which is the case with compounds **10c** and **5c** .

**Table 5:** Ionization potential ( *I* ), electron affinity ( *A* ), chemical hardness (η), electronic chemical potential (µ), and electrophilicity index (ω) of the compounds under study. As per Koopman’s theorem, the negative of E _*HOMO*_ and E _*LUMO*_ correspond to the ionization potential ( *I* ) and electron affinity ( *A* ) of the compound. The other descriptors, i.e. chemical hardness (η), electronic chemical potential (µ), and electrophilicity index (ω), have been calculated as given in the table.

Compound	Ionization potential, *I* (eV)	Electronaffinity, *A* (eV)	Chemical hardness,ƞ (eV) η = (E _*HOMO*_ - E _*LUMO*_ )/2	Electronic chemical potential,μ (eV) η = - (E _*HOMO*_ + E _*LUMO*_ )/2	Electrophilicity index,ω (eV) ω = μ2/2η
**5a**	6.59	1.90	2.34	–4.24	3.83
**5b**	6.60	1.97	2.32	–4.28	3.96
**5c**	5.85	1.72	2.07	–3.79	3.47
**5d**	6.56	1.89	2.34	–4.22	3.82
**5e**	6.57	2.00	2.29	–4.28	4.01
**10a**	6.61	1.91	2.35	–4.26	3.87
**10b**	6.63	2.24	2.19	–4.43	4.48
**10c**	5.87	1.75	2.06	–3.81	3.52
**10d**	6.59	2.02	2.29	–4.31	4.05
**10e**	6.58	1.91	2.34	–4.25	3.86
**10f**	6.47	1.90	2.29	–4.18	3.83

## 3. Experimental analysis

A Buchi B-540 was used as a melting point apparatus to determine the melting point of the newly synthesized compounds. The analytical grade chemicals and reagents were acquired from Alfa Aesar and Sigma Aldrich. To measure the _1_ H NMR and _13_ C NMR spectra of synthesized products at 400 MHz in the presence of CDCl _3_ , the instrument Avance III Bruker spectrometer was used. The coupling constant values were measured in Hertz, while the chemical shift (δ) data were determined in ppm. EI-MS data were obtained using a JMS-HX-110 spectrometer while a Thermo Finnigan FlasH _11_ 12 apparatus was used for elemental analysis. For the purification of the compounds, column chromatography with silica gel was used. The reaction completion was confirmed with the help of thin-layer chromatography (TLC) on the cards having silica gel (pore size 60 A° and coated with PF _254_ , Merck, Germany). The spots produced by newly synthesized thiophene derivatives were detected by a UV lamp (254 to 365 nm).

### 3.1. Synthesis of the pentyl 5-bromothiophene-2-carboxylate (3)

To carry out the esterification, a weighed amount of **1** (4 g, 19.0 mmol) was taken in a dried flask (250 mL) along with DCM (200 mL) solvent and a magnetic stirrer. The amyl alcohol (3 equiv.) and DMAP (0.05 equiv.) were added to the above mixture and maintained at 0 °C. After 10 min., the coupling reagent DCC (1.1 equiv.) was added to it and stirred. After 15 min., the round bottom flask was taken out from isotherm and shifted at 30 °C. It was then stirred for about 6 h; and after that, the filtration of the mixture and evaporation of the solvent from filtrate were performed. Soon afterwards, the final product was purified by using column chromatography, and a mixture of n-hexane (90%) and ethyl acetate (10%) was used as the eluent. Finally, the NMR techniques were applied to find out the purity and structure of the newly synthesized compound [21].

### 3.1.1. Pentyl 5-bromothiophene-2-carboxylate (3)

A yellowish solid. mp: 161.1 °C. C _10_ H _13_ BrO _2_ S (requires: C, 43.32; H, 4.72; found: C, 43.29; H, 4.68%). EI-MS *m/z* (+ion mode): 277.16: [M – Br] ^+^ = 197.26: [M – C _5_ H _11_ ] ^+^ = 125.97: [M – CO _2_ ] ^+^ = 81.98. IR (KBr): 3028, 2955, 1725, 1590, 1284, 1115, 724 cm ^-1^ . _1_ H NMR (CDCl _3_ ): *δ* = 7.55 (d, *J* = 7.48, 1H-Thiophene), 7.01 (d, *J* = 7.41, 1H-Thiophene), 4.27 (t, *J* = 7.02, 2H-CH _2_ ), 1.75 (m, 2H-CH _2_ ), 1.51 (m, 2H-CH _2_ ), 1.41 (m,2H-CH _2_ ), 0.91 (t, *J* = 8.01, 3H-CH _3_ ). _13_ C NMR (CDCl _3_ ): *δ* = 162.12, 136.22, 135.10, 131.25, 122.20, 64.11, 29.10, 28.19, 22.11, 14.10.

### 3.2. General procedure for the synthesis of compounds **5a** - **5e**

To a dried schlenk flask, a weighted amount of catalyst Pd(PPh _3_ ) _4_ (5 mol%), solvent (5 mL), and 3 (0.17 g, 0.61 mmol) were taken in an inert atmosphere and stirred for half an hour. To the above mixture, the base K3PO4 (1.28 mmol) and arylboronic acid (0.67 mmol) were added. It was then refluxed under an inert atmosphere for about 16 h at 90 °C. The reaction completion was confirmed by TLC. The n-hexane (80-90%) and ethyl acetate (10-20%) were both used as a solvent mixture for TLC. The temperature was then lowered to 25 °C, and the mixture was filtered and concentrated by evaporating the solvent from filtrate with the help of a rotary evaporator. The column chromatography was employed for the purification of the synthesized products. For this purpose, a mixture of n-hexane (70%–85%) and ethyl acetate (15%–30%) was taken as eluent. Later on, the newly synthesized compounds were purified and then analyzed by using NMR spectroscopy [52].

### 3.2.1. Pentyl 5-(3-chlorophenyl)thiophene-2-carboxylate ( **5a** )

A yellowish solid. mp: 232.7 °C. C _16_ H _17_ ClO _2_ S (requires: C, 62.22; H, 5.55; found: C, 62.19; H, 5.47%). EI-MS *m/z* (+ion mode): 308.06: [M – Cl] ^+^ = 273.07: [M – C _5_ H _11_ ] ^+^ = 202.03: [M – CO _2_ ] ^+^ = 158.01: [M – C _6_ H _4_ ] ^+^ = 82.0. IR (KBr): 3031, 2950, 1722, 1588, 1472, 1285, 1118, 790, 742, 723 cm ^-1^ . _1_ H NMR (CDCl _3_ ): *δ* = 7.75 (t, *J* = 3.7 Hz, 1H-Ph), 7.61 (d, *J* = 0.7 Hz, 1H-Ph), 7.52–7.49 (m, 1H-Ph), 7.35–7.29 (m, 2H-Thiophene), 7.29–7.28 (m, 1H-Ph), 4.34 (t, *J* = 6.7 Hz, 2H-CH _2_ ), 1.85–1.73 (m, 1H-CH _2_ ), 1.65 (q, *J* = 6.8 Hz, 2H-CH _2_ ), 1.59 (s, 1H-CH _2_ ), 0.98 (d, *J* = 6.6 Hz, 5H-C _2_ H5). _13_ C NMR (CDCl _3_ ): *δ* = 162.45, 145.87, 135.90, 134.95, 134.01, 133.97, 130.01, 129.53, 128.58, 127.10, 124.15, 64.21, 29.11, 28.21, 22.56, 14.19.

### 3.2.2. Pentyl 5-(3-acetylphenyl)thiophene-2-carboxylate ( **5b** )

A colorless solid. mp: 275.3 °C. C _18_ H _20_ O _3_ S (requires: C, 68.32; H, 6.36; found: C, 68.29; H, 6.31%). EI-MS *m/z* (+ion mode): 316.10: [M – COCH _3_ ] ^+^ = 273.10: [M – C _5_ H _11_ ] ^+^ = 202.01: [M – C _6_ H _4_ ] ^+^ = 125.97: [M – CO _2_ ] ^+^ = 81.97. IR (KBr): 3029, 2985, 2952, 1722, 1705, 1590, 1470, 1282, 1121, 792, 723 cm ^-1^ . _1_ H NMR (CDCl _3_ ): *δ* = 8.21 (s, 1H-Ph), 7.92 (d, *J* = 7.8 Hz, 1H-Thiophene), 7.82 (d, *J* = 8.5 Hz, 1H-Thiophene), 7.77 (t, *J* = 3.7 Hz, 1H-Ph), 7.51 (t, *J* = 7.8 Hz, 1H-Ph), 7.39–7.33 (m, 1H-Ph), 4.34 (t, *J* = 6.7 Hz, 2H-CH _2_ ), 2.65 (s, 3H-CH _3_ ), 1.86–1.74 (m, 1H-CH _2_ ), 1.66 (dd, *J* = 13.7, 6.9 Hz, 3H-CH _2_ ), 0.98 (d, *J* = 6.6 Hz, 5H-C _2_ H5). _13_ C NMR (CDCl _3_ ): *δ* = 196.95, 162.12, 146.45, 137.11, 135.90, 134.35, 133.01, 130.04, 129.87, 128.96, 128.01, 126.15, 64.31, 29.14, 28.35, 26.67, 22.49, 14.23.

### 3.2.3. Pentyl 5-(4-(methylthio)phenyl)thiophene-2-carboxylate ( **5c** )

A colorless solid. mp: 248.4–250.2 °C. C _17_ H _20_ O _2_ S _2_ (requires: C, 67.72; H, 6.28; found: C, 67.66; H, 6.25%). EI-MS *m/z* (+ion mode): 320.08: [M – SCH _3_ ] ^+^ = 273.06: [M – C _5_ H _11_ ] ^+^ = 202.04: [M – CO _2_ ] ^+^ = 158.02: [M – C _6_ H _4_ ] ^+^ = 81.99. IR (KBr): 3025, 2980, 2945, 1719, 1591, 1471, 1279, 1121, 789, 725, 708 cm ^-1^ . _1_ H NMR (CDCl _3_ ): *δ* = 7.79–7.67 (m, 2H-Ph), 7.54 (d, *J* = 8.3 Hz, 2H-Thiophene), 7.24 (d, *J* = 4.0 Hz, 2H-Ph), 4.33 (t, *J* = 6.8 Hz, 2H-CH _2_ ), 2.50 (s, 3H-CH _3_ ), 1.85–1.73 (m, 1H-CH _2_ ), 1.65 (q, *J* = 6.5 Hz, 2H-CH _2_ ), 0.97 (d, *J* = 6.6 Hz, 6H- CH _2_ -CH _3_ ). _13_ C NMR (CDCl _3_ ): *δ* = 162.32, 146.23, 139.11, 135.09, 134.09, 130.25, 129.10, 127.98, 126.75, 64.27, 28.95, 28.01, 22.03, 14.92, 14.02.

### 3.2.4. Pentyl 5-(3-chloro-4-fluorophenyl)thiophene-2-carboxylate( **5d** )

A white solid. mp: 245.8 °C. C _16_ H _16_ ClFO _2_ S (requires: C, 58.79; H, 4.92; found: C, 58.75; H, 4.88%). EI-MS *m/z* (+ion mode): 326.04: [M – FCl] ^+^ = 272.08: [M – C _5_ H _11_ ] ^+^ = 202.05: [M – CO _2_ ] ^+^ = 158.03: [M – C _6_ H _4_ ] ^+^ = 81.98. IR (KBr): 3030, 2949, 1720, 1592, 1475, 1281, 1122, 1090, 791, 743, 726 cm ^-1^ . _1_ H NMR (CDCl _3_ ): *δ* = 7.74 (t, *J* = 3.7 Hz, 1H-Ph), 7.65 (dd, *J* = 6.8, 2.2 Hz, 1H-Thiophene), 7.49–7.46 (m, 1H-Thiophene), 7.22–7.15 (m, 2H-Ph), 4.34 (t, *J* = 6.7 Hz, 2H-CH _2_ ), 1.85–1.73 (m, 1H-CH _2_ ), 1.65 (dd, *J* = 13.6, 6.8 Hz, 2H-CH _2_ ), 0.98 (t, *J* = 6.3 Hz, 6H-CH _2_ -CH _3_ ). _13_ C NMR (CDCl _3_ ): *δ* = 162.45, 158.11, 146.43, 135.01, 134.16, 130.05, 129.02, 128.96, 127.25, 121.10, 117.12, 64.11, 28.95, 28.01, 22.05, 14.50.

### 3.2.5. Pentyl 5-(3,4-dichlorophenyl)thiophene-2-carboxylate( **5e** )

A colorless solid. mp: 275.2 °C. C _16_ H _16_ Cl _2_ O _2_ S (requires: C, 55.97; H, 4.69; found: C, 55.93; H, 4.65%). EI-MS *m/z* (+ion mode): 343.26: [M – 2Cl] ^+^ = 272.06: [M – C _5_ H _11_ ] ^+^ = 201.01: [M – CO _2_ ] ^+^ = 157.02: [M – C _6_ H3] ^+^ = 81.97. IR (KBr): 3028, 2955, 1720, 1595, 1470, 1279, 1121, 788, 735, 725 cm ^-1^ . _1_ H NMR (CDCl _3_ ): *δ* = 7.75 (d, *J* = 3.7 Hz, 1H-Ph), 7.70 (d, *J* = 1.8 Hz, 1H-Ph), 7.48–7.42 (m, 2H-Thiophene), 7.27 (d, *J* = 4.2 Hz, 1H-Ph), 4.34 (t, *J* = 6.7 Hz, 2H-CH _2_ ), 1.85–1.73 (m, 1H-CH _2_ ), 1.67–1.62 (m, 3H-CH _2_ ), 0.97 (d, *J* = 6.6 Hz, 5H-C _2_ H5). _13_ C NMR (CDCl _3_ ): *δ* = 162.41, 146.10, 135.02, 134.05, 133.45, 132.91, 132.02, 130.09, 129.15, 128.11, 127.13, 64.11, 28.61, 27.95, 22.15, 14.29.

### 3.3. Synthesis of the phenethyl 5-bromothiophene-2-carboxylate (7)

To carry out the esterification again, a weighed amount of 1 (6 g, 29.0 mmol) was taken in the dried flask (250 mL) along with DCM (200 mL) solvent and a magnetic stirrer. The 2-phenylethanol (3 equiv.) and DMAP (0.05 equiv.) were added to the above mixture and the mixture was kept at 0 °C. After 10 min., the coupling reagent DCC (1.1 equiv.) was added to it and stirred for 15 min. Then, the round bottom flask was taken out from isotherm and shifted at 30 °C. It was then stirred for about 6 h; and after that, the filtration of mixture and evaporation of the solvent from filtrate were performed. Soon afterwards, the final product was purified by using column chromatography. For this, a solvent mixture of n-hexane and ethyl acetate was used as the eluent in the ratio of 85% and 15%, respectively. Finally, the products were confirmed through 1H and _13_ C NMR spectral data [21].

### 3.3.1. Phenethyl 5-bromothiophene-2-carboxylate (7)

A yellowish compound. mp: 489.4 °C. C _13_ H _11_ BrO _2_ S (requires: C, 50.16; H, 3.56; found: C, 50.12; H, 3.47%). EI-MS *m/z* (+ion mode): 311.89: [M – Br] ^+^ = 231.04: [M – C _6_ H5] ^+^ = 154.02: [M – C _3_ H _4_ O _2_ ] ^+^ = 81.98.IR (KBr): 3028, 2950, 1725, 1595, 1500, 1281, 1120, 1052, 724 cm ^-1^ . _1_ H NMR (CDCl _3_ ): *δ* = 7.76 (d, *J* = 7.46, 1H-Thiophene), 7.38 (dd, *J* = 7.47, 1.48, 2H-Ph), 7.27 (dd, *J* = 7.47, 1.48, 2H-Ph), 7.21 (t, *J* = 7.46, 1H-Ph), 6.98 (d, *J* = 7.48, 1H-Thiophene), 4.51 (t, *J* = 7.08, 2H-CH _2_ ) 3.01 (t, *J* = 7.04, 2H-CH _2_ ). _13_ C NMR (CDCl _3_ ): *δ* = 162.68, 138.02, 136.78, 135.60, 131.58, 129.02, 127.98, 125.88, 122.06, 64.10, 34.10.

### 3.4. General procedure for the synthesis of compounds **10a** - **10f**

A schlenk flask was taken and dried in an oven, and a magnetic stirrer was put inside it. In the present flask, phenethyl 5-bromothiophene-2-carboxylate 3 (0.26 g, 0.64 mmol), the solvent (5 mL) and tetrakis (triphenylphosphine) palladium (0) were taken and stirred for half an hour at 25 °C. After that, the arylboronic acid (0.64 mmol) and K3PO4 (1.28 mmol) were added, and the reaction mixture was refluxed at 90 °C for 16 h. After the completion of the reaction, the temperature was lowered to room temperature and the mixture was filtered. The excess solvent present in the solution was evaporated by a rotary evaporator. The column chromatography was used to purify the desired products, and a mixture of n-hexane (65%–85%) and ethyl acetate (15%–35%) was taken as the eluent. The product was desiccated, and the structures were characterized through NMR spectral data [3].

### 3.4.1. Phenethyl 5-(3-chlorophenyl)thiophene-2-carboxylate ( **10a** )

A white solid. mp 566.1 °C. C _19_ sub>H _15_ ClO _2_ S (requires: C, 66.55; H, 4.40; found: C, 66.51; H, 4.37%). EI-MS *m/z* (+ion mode): 342.03: [M – Cl] ^+^ = 307.07: [M – C _6_ H _4_ ] ^+^ = 231.04: [M – C _6_ H5] ^+^ = 154.03: [M – C _3_ H _4_ O _2_ ] ^+^ = 81.97.IR (KBr): 3029, 2952, 1725, 1591, 1502, 1475, 1279, 1121, 1052, 792, 744, 725 cm ^-1^ . _1_ H NMR (CDCl _3_ ): *δ* = 7.73 (d, *J* = 3.3 Hz, 1H-Ph), 7.56 (d, *J* = 7.4 Hz, 2H-Thiophene), 7.38 (d, *J* = 7.5 Hz, 2H-Ph), 7.35–7.25 (m, 6H-Ph), 4.51 (t, *J* = 6.9 Hz, 2H-CH _2_ ), 3.07 (t, *J* = 6.9 Hz, 2H-CH _2_ ). _13_ C NMR (CDCl _3_ ): *δ* = 162.88, 146.12, 138.24, 136.01, 135.04, 134.84, 134.02, 130.04, 129.45, 128.90, 127.50, 126.95, 125.60, 124.12, 123.96, 64.75, 34.30.

### 3.4.2. Phenethyl 5-(3-acetylphenyl)thiophene-2-carboxylate ( **10b** )

A yellowish solid. mp: 608.7 °C. C _21_ H _18_ O _3_ S (requires: C, 71.96; H, 5.17; found: C, 71.91; H, 5.14%). EI-MS *m/z* (+ion mode): 350.08: [M – COCH _3_ ] ^+^ = 307.09: [M – C _6_ H _4_ ] ^+^ = 231.03: [M – C _6_ H5] ^+^ = 154.01: [M – C _3_ H _4_ O _2_ ] ^+^ = 81.96.IR (KBr): 3030, 2995 2952, 1723, 1705, 1597, 1502, 1474, 1282, 1124, 1050, 791, 724 cm ^-1^ . _1_ H NMR (CDCl _3_ ): *δ* = 8.48 (s, 1H-Ph), 8.01 (dd, *J* = 7.47, 1.48, 1H-Ph), 7.90 (dd, *J* = 7.51, 1.49, 1H-Ph), 7.82 (d, *J* = 7.52, 1H-Thiophene), 7.74 (d, *J* = 7.48, 1H-Thiophene), 7.60 (t, *J* = 7.52, 1H-Ph), 7.39 (t, *J* = 7.51, 2H-Ph), 7.29 (dd, *J* = 7.45, 1.48, 2H-Ph), 7.21 (t, *J* = 7.48, 1H-Ph), 4.49 (t, *J* = 7.11, 2H-CH _2_ ) 3.02 (t, *J* = 7.09, 2H-CH _2_ ), 2.51 (s, 3H-CH _3_ ). _13_ C NMR (CDCl _3_ ): *δ* = 196.96, 162.79, 146.21, 139.01, 137.03, 135.01, 134.10, 133.65, 130.32, 129.50, 129.01, 128.90, 128.47, 127.45, 126.05, 125.88, 64.81, 34.52, 26.34.

### 3.4.3. Phenethyl 5-(3-(methylthio)phenyl)thiophene-2-carboxylate ( **10c** )

A colorless solid. mp: 581.9 °C. C _2_ 0H _18_ O _2_ S _2_ (requires: C, 67.75; H, 5.11; found: C, 67.71; H, 5.07%). EI-MS *m/z* (+ion mode): 354.06:[M – SCH _3_ ] ^+^ = 307.06: [M – C _6_ H _4_ ] ^+^ = 231.01: [M – C _6_ H5] ^+^ = 154.0: [M – C _3_ H _4_ O _2_ ] ^+^ = 81.99.IR (KBr): 3025, 2988, 2950, 1721, 1595, 1500, 1472, 1283, 1121, 1049, 792, 723, 711 cm ^-1^ . _1_ H NMR (CDCl _3_ ): *δ* = 7.85 (d, *J* = 7.44, 1H-Thiophene), 7.78 (d, *J* = 7.42, 1H-Thiophene), 7.65 (dd, *J* = 7.42, 1.47, 2H-Ph), 7.47 (dd, *J* = 7.45, 1.46, 2H-Ph), 7.37 (t, *J* = 7.41, 2H-Ph), 7.31 (dd, *J* = 7.44, 1.48, 2H-Ph), 7.22 (t, *J* = 7.46, 1H-Ph), 4.55 (t, *J* = 7.07, 2H-CH _2_ ), 2.99 (t, *J* = 7.11, 2H-CH _2_ ), 2.51 (s, 3H-CH _3_ ). _13_ C NMR (CDCl _3_ ): *δ* = 161.89, 145.67, 138.91, 138.01, 135.02, 134.42, 130.12, 129.56, 128.97, 127.61, 126.71, 125.56, 124.67, 64.69, 34.35, 14.77.

### 3.4.4. Phenethyl 5-(3,4-dichlorophenyl)thiophene-2-carboxylate ( **10d** )

A colorless solid. mp: 608.5 °C. C _19_ H _14_ Cl _2_ O _2_ S (requires: C, 60.48; H, 3.74; found: C, 60.43; H, 3.67%). EI-MS *m/z* (+ion mode): 376.01:[M – 2Cl] ^+^ = 306.06: [M – C _6_ H3] ^+^ = 231.04: [M – C _6_ H5] ^+^ = 154.02: [M – C _3_ H _4_ O _2_ ] ^+^ = 81.97.IR (KBr): 3028, 2952, 1723, 1594, 1501, 1476, 1281, 1122, 1052, 789, 738, 723 cm ^-1^ . _1_ H NMR (CDCl _3_ ): *δ* = 7.73 (d, *J* = 3.9 Hz, 1H-Ph), 7.66 (dd, *J* = 6.8, 2.3 Hz, 1H-Thiophene), 7.50–7.46 (m, 1H-Thiophene), 7.35–7.16 (m, 7H-Ph), 4.51 (t, *J* = 7.0 Hz, 2H-CH _2_ ), 3.07 (t, *J* = 7.0 Hz, 2H-CH _2_ ). _13_ C NMR (CDCl _3_ ): *δ* = 163.02, 145.98, 138.59, 135.17, 134.45, 133.70, 133.11, 132.15, 131.11, 129.90, 129.48, 128.93, 127.89, 126.92, 126.07, 65.11, 34.90.

### 3.4.5. Phenethyl 5-(4-chloro-3-fluorophenyl)thiophene-2-carboxylate ( **10e** )

A yellowish solid. mp: 579.2 °C. C _19_ H _14_ ClFO _2_ S (requires: C, 63.23; H, 3.90; found: C, 63.20; H, 3.86%). EI-MS *m/z* (+ion mode): 360.02: [M – FCl] ^+^ = 306.05: [M – C _6_ H3] ^+^ = 231.01: [M – C _6_ H5] ^+^ = 154.04: [M – C _3_ H _4_ O _2_ ] ^+^ = 81.99. IR (KBr): 3031, 2955, 1720, 1595, 1500, 1477, 1279, 1120, 1088, 1051, 791, 743, 724 cm ^-1^ . _1_ H NMR (CDCl _3_ ): *δ* = 7.73 (d, *J* = 3.9 Hz, 1H-Ph), 7.66 (dd, *J* = 6.5, 1.8 Hz, 1H-Thiophene), 7.51–7.43 (m, 1H-Thiophene), 7.38–7.14 (m, 7H-Ph), 4.51 (t, *J* = 7.0 Hz, 2H-CH _2_ ), 3.07 (t, *J* = 7.0 Hz, 2H-CH _2_ ). _13_ C NMR (CDCl _3_ ): *δ* = 162.79, 157.92, 146.34, 138.35, 135.10, 134.05, 130.86, 129.85, 129.13, 128.88, 127.91, 126.89, 125.86, 121.18, 117.38, 64.67, 34.58.

### 3.4.6. Phenethyl 5-(4-chlorophenyl)thiophene-2-carboxylate ( **10f** )

A yellowish solid. mp: 452.8 °C. C _19_ H1 **5c** lO _2_ S (requires: C, 66.55; H, 4.40; found: C, 66.47; H, 4.37%). EI-MS *m/z* (+ion mode): 342.05: [M – Cl] ^+^ = 307.05: [M – C _6_ H _4_ ] ^+^ = 231.02: [M – C _6_ H5] ^+^ = 154.01: [M – C _3_ H _4_ O _2_ ] ^+^ = 81.98. IR (KBr): 3029, 2954, 1719, 1595, 1502, 1478, 1282, 1125, 1055, 791, 750, 724 cm ^-1^ . _1_ H NMR (CDCl _3_ ): *δ* = 7.90 (d, *J* = 7.47, 1H-Thiophene), 7.79 (d, *J* = 7.51, 1H-Thiophene), 7.68 (dd, *J* = 7.49, 1.48, 2H-Ph), 7.53 (dd, *J* = 7.46, 1.45, 2H-Ph), 7.42 (t, *J* = 7.52, 2H-Ph), 7.30 (dd, *J* = 7.50, 1.49, 2H-Ph), 7.25 (t, *J* = 7.51, 1H-Ph), 4.51 (t, *J* = 7.05, 2H-CH _2_ ) 2.95 (t, *J* = 7.02, 2H-CH _2_ ). _13_ C NMR (CDCl _3_ ): *δ* = 162.81, 146.05, 138.11, 135.23, 134.93, 134.31, 131.77, 130.45, 129.90, 128.96, 128.42, 127.83, 126.82, 64.71, 34.41.

### 3.5. General procedure for the spasmolytic activity

To examine the smooth muscle relaxant or spasmolytic potential of the synthesized compounds, isolated rat duodenum was used. Prior to the experimentation, the rats were kept on starvation for 24 h and then sacrificed by a blow on the head, and subsequently the duodenum was removed. It was cleaned of the mesenteries and was cut into small pieces having a size of 2-3 cm. The tissue was hanged into tissue organ bath containing Tyrode’s solution with a continuous supply of carbogen, and a tension of 1 g was applied to the tissue. The tissue was allowed to stabilize in the organ bath up to 20–30 min. before the addition of any drug/compound. Then, it was equilibrated with repeated exposure of acetylcholine (1 µM). After that, equilibration tissue was exposed to K ^+^ -80 (High-K), which induced contractions. After attaining the sustained induced contractions, the tissue was exposed to test compound cumulatively in a dose-dependent fashion, responses were recorded by isotonic transducers (MLT 0015, Panlab, Spain) connected to the PowerLab data acquisition system (AD Instruments, Australia). The effect was measured as the percentage of the induced contractions just before the administration of the test material [23,53–55].

## 4. Conclusion

In this work hereby, thiophene-based derivatives ( **5a** - **5e** , **10a** - **10f** ) were synthesized by the Suzuki cross-coupling reaction of pentyl 5-bromothiophene-2-carboxylate (3) and phenethyl 5-bromothiophene-2-carboxylate (7) with several arylboronic acids in the presence of Pd(PPh _3_ ) _4_ and K3PO4. Compounds 3 and 7 were synthesized from commercially available compound 5-bromothiophene-2-carboxylic acid via Steglich esterification reaction. The thiophene-based derivatives ( **5a** - **5e** , **10a** - **10f** ) were synthesized from the reaction of 3 and 7 by the application of the Suzuki cross-coupling reaction. The spasmolytic activity of all the synthesized compounds was investigated to study the biological significance of these compounds. The results revealed that compound **10d** showed the highest potency with EC _50_ value 1.26. Our recent findings suggest that thiophene-based derivatives inhibit the contractile responses of the rat duodenum. The results from the present study have shown that the spasmolytic activity of thiophene derivatives may be due to the blockage of calcium channels since thiophene derivatives possessed calcium channel blocking activity. The replacement of the hydroxyl group with the phenyl group may enhance the spasmolytic activity of thiophene derivatives. This blockage of calcium channels is receptor linked or direct, and requires highly sophisticated and extensive research. The synthesized thiophene derivatives have been studied using DFT calculations for their structural and electronic properties. A detailed insight into FMOs of the compounds and their different reactivity descriptors revealed that compounds **10c** and **5c** are the most reactive while **10a** is the most stable in the series. **10c** and **5c** also showed a very good NLO response with the highest β values.
